# High‐sensitive clinical diagnostic method for PTPRZ1‐MET and the characteristic protein structure contributing to ligand‐independent MET activation

**DOI:** 10.1111/cns.13627

**Published:** 2021-02-28

**Authors:** Ruoyu Huang, Yanwei Liu, Kuanyu Wang, Zheng Wang, Chuanbao Zhang, Wei Zhang, Zheng Zhao, Guanzhang Li, Lijie Huang, Yuanhao Chang, Fan Zeng, Tao Jiang, Huimin Hu

**Affiliations:** ^1^ Department of Molecular Neuropathology Beijing Neurosurgical Institute Capital Medical University Beijing China; ^2^ Department of Neurosurgery Beijing Tiantan Hospital Capital Medical University Beijing China; ^3^ Center of Brain Tumor Beijing Institute for Brain Disorders Beijing China; ^4^ China National Clinical Research Center for Neurological Diseases Beijing China; ^5^ Chinese Glioma Genome Atlas Network (CGGA) Beijing China

**Keywords:** coiled‐coil structure, glioma progression, MET inhibitor, receptor tyrosine kinase

## Abstract

**Background:**

*PTPRZ1*‐*MET* (*ZM*) is a critical genetic alteration driving the progression of lower‐grade glioma. Glioma patients harboring *ZM* could benefit from MET inhibitors. According to the remarkable role of *ZM* as a driver of glioma progression and indicator of MET inhibitor sensitivity, it is necessary to detect this alteration even when it presents in glioma with relatively fewer copies.

**Methods:**

Herein, we proposed that *ZM* could be detected with a high‐sensitive method of reverse transcriptase PCR with 50 amplification cycles. Via this newly proposed detection method, we depicted the incidence preference of *ZM* fusion in a cohort of 485 glioma patients. To further explore the oncogenic nature of *ZM*, we predicated the protein structure alteration of MET kinase brought by its fusion partner.

**Results:**

The incidence of *ZM* fusions was much higher than previous report. *ZM* fusions exhibited a striking preference in lower‐grade glioma and secondary glioblastoma. By contrast, none of patients with primary glioblastoma was detected harboring *ZM* fusion. In each of the four variants of ZM, the fusion partner segment of MET contained a remarkable coiled‐coil motif. In glioma cells expressing *ZM*, MET kinase could be activated in a ligand‐independent manner, which might be contributed by the special coiled‐coil structure brought by the fusion partner. Corresponding to the 3D structural analysis and cell line experiment, the *ZM* positive clinical specimens showed hyperactivations of MET signaling.

**Conclusions:**

*ZM* fusions are critical drivers of glioma progression and effective target of MET inhibitor. Early detection could be performed with a high‐sensitive method of reverse transcriptase PCR. The hyperactivations of MET signaling driving glioma progression might be contributed by a ligand‐independent activation enabled by the protein structure modification of extracellular domain of MET in ZM fusions.

## Graphical abstract

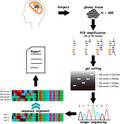

## 1 INTRODUCTION

Gliomas including lower‐grade glioma (LGG, World Health Organization grades II and III) and glioblastoma (GBM, WHO IV)^1^ are highly fatal primary intracranial tumor. Although patients of LGGs live longer with symptoms of less severity, their cancers will progress into secondary glioblastoma (sGBM) invariably. Unlike that the static states of LGG keep for 5–10 years, sGBM progresses rapidly and the median overall survival of patients with sGBM is only 31 months even after surgery and adjuvant chemoradiotherapy.^2^ With paired LGG and sGBM samples, several longitudinal studies proposed the roles of PI3 K/Akt/mTOR pathway, RB pathway,^3^, ^4^ cell‐cycle regulations,^5^ and DNA methylation reprogramming^6^ in driving or at least contributing to glioma progression. In the resent research of our laboratory, we illustrated the driver role of a fusion gene, *PTPRZ1*‐*MET* (*ZM*), in the progression of LGG to sGBM.^7^ We had known that *ZM* happens in ~14% of cases of sGBM with significantly worse prognosis and causes activation of MET signaling.^7^ The abnormal MET activation led to glioma progression and indicated MET‐inhibition sensitivity. These findings guided to a clinical trial of treatment of patients with high‐grade glioma (WHO III & IV) with novel MET‐targeted compound. Tumor shrinkage and symptom relief were observed after treatment. The trial of clinical phase II is ongoing.

As *ZM* is a driver of glioma progression and an indicator of MET‐inhibition sensitivity, it is meaningful to detect *ZM* fusions even when they are still present in tumors with relative fewer copies. Herein, we report a high‐sensitive and low‐cost method to detect *ZM* fusions in glioma tissues. This method could be used as the “gold standard” of *ZM* positive (*ZM*+) glioma diagnosis as the fusion point of *PTPRZ1* and *MET* could be confirmed via Sanger sequencing included in this method. Despite the critical roles of *ZM* fusions in LGG progression that have been proposed, the intrinsic mechanism of MET activation launched by *ZM* has not been explored. With 3D protein structure predication, we found that the fusion partner fragment added to MET kinase in ZM fusion protein contains a coiled‐coil structure which might contribute to the ligand‐independent MET activation.

## 2 MATERIALS AND METHODS

### 2.1 Human tissues and dataset

Glioma tissues and the patients’ follow‐up information (diagnosis, gender, age, WHO grade, PFS, and OS) were obtained from the Chinese Glioma Genome Atlas (CGGA) dataset. Patients treated at Beijing Tiantan Hospital, Sanbo Hospital in Beijing, Harbin Medical University and China Medical University were included in this study. Written consent was obtained from all patients. The glioma tissues were snap‐frozen in liquid nitrogen immediately after surgical resection and preserved in liquid nitrogen. Only samples with >80% tumor cells were enrolled for analysis. The establishment and management of our CGGA databank has been introduced in our previous publications.^8^, ^9^ Information of the critical histology and molecular features of the tumors in this study was derived from CGGA dataset (http://www.cgga.org.cn/).

The peripheral blood from patients harboring *ZM*+ or *ZM* negative (*ZM*−) glioma used in this study was collected from dorsalis pedis artery. Two milliliters of whole blood were collected from each patient.

All the processes of tissues and information collection were approved by the Institutional Review Boards of Beijing Tiantan Hospital (No. KY2014‐002‐02) and were conducted in accordance with the principles expressed in the Declaration of Helsinki.

### 2.2 Detection *ZM* fusions in glioma tissues and peripheral blood

Glioma tissue RNA was abstracted using the RNAprep pure Tissue Kit (Tiangen Biotech) followed by cDNA synthesis (RevertAid RT Kit, Thermo Fisher Scientific).

RNA concentration and quality were measured using the NanoDrop ND‐1000 spectrophotometer (NanoDrop Technologies). Only RNA with A260/A280 values >1.8 and <2.0 were adopted in following processes. The integrity of total RNA was evaluated by agarose gel electrophoresis after the RNA samples were ten times diluted. Only if the bands of 28S rRNA and 18S rRNA were both bright and clear, and the bright intensity of 28S rRNA: 18S rRNA was about 2:1, the RNA was identified as qualified. Complementary DNA was synthesized from 1 μg of qualified total RNA using the SuperScript III First‐Strand Synthesis SuperMix kit (Invitrogen).

RT‐PCR were performed using 1 μl of cDNA. The PCR program included denaturing at 95°C (30 s), annealing at 58°C (30 s), and extension at 72°C (30 s). Primers flanking the fusion points of *ZM* (forward: 5′‐CCGTCTGGAAATGCGAATCCTAAA‐3′ reverse: 5′‐CAGGCCCAGTCTTGTACTCAGCAA‐3′) were used to clone the sequences crossing the fusion points with DNA polymerase (GoTaq, Promega). The primers were designed based on the *PTPRZ1* and *MET* segments that are common to all the four ZM fusion variants. All the four *ZM* fusion variants could be specifically amplified using this pair of primers, and amplification products were of different sizes (*ZM* variant 1: 337 bp; *ZM* variant 2: 403 bp; *ZM* variant 3: 583 bp; *ZM* variant 4: 1207 bp). In every examination, a negative control test to avoid cross‐contamination or false positive was performed simultaneously with nuclease‐free water, and a positive control test to avoid false negative was performed with an already‐known *ZM*+ cDNA.

After electrophoresis, the amplification product bands were purified and submitted for Sanger sequencing. The sequencing reads were aligned to the known *ZM* fusion sequences to determine whether *ZM* fusions were present in the given glioma specimen and which variant it was.

RNA of whole blood was abstracted with RNAprep Pure Hi‐Blood Kit (Tiangen Biotech). The following reverse transcription step and *ZM* fusions examination step were as the corresponding steps in *ZM* detection of glioma tissues mentioned above.

Quantification of the bands’ gray intensity was performed with the software of ImageJ following phase inversion.

### 2.3 Cell culture

Glioma cell line U87 MG was obtained from the Institute of Biochemistry and Cell Biology, Chinese Academy of Sciences, and was cultured in Dulbecco's Modified Eagle's Medium (DMEM) with 4.5 g/L glucose. The DMEM culture medium was supplemented with 10% fetal bovine serum (FBS, HyClone), 100 units/ml penicillin, and 100 μg/ml streptomycin (Invitrogen). The cell line was cultured at 37°C in a humidified atmosphere of 5% CO_2_.

### 2.4 Construction of adenoviral vectors encoding *ZM* fusions and transduction *ZM* variants into glioma cell line

*ZM* variant 1 and *ZM* variant 2 coding sequences were separately cloned from *ZM*+ gliomas tissues and transferred into a His6‐tagged pShuttle‐CMV vector. The sequences were then individually recombined into the pADxsi vector. Insertion of the right sequences was confirmed with restriction enzyme digestion and sequencing. The recombinant plasmid was subsequently linearized by restriction enzyme digestion and transfected into HEK293A cells with Lipofectamine2000 (Thermo Fisher Scientific) for the generation of adenoviral vectors. The collected adenoviral vector was aliquoted and stored at −80°C before use. U87 MG cells were treated in the medium containing an appropriate titer of the adenoviral vector for 6 h before medium change. *ZM* fusions expression in the cell line was confirmed by RT‐PCR.

### 2.5 Western blotting

Glioma tissue or U87 MG cells were lysed with RIPA buffer (Cell Signaling Technology) supplemented with protease inhibitor (Solarbio biotech). Protein concentration was evaluated using Coomassie Brilliant Blue on a microplate spectrophotometer (Infinite M200 PRO, Tecan). Equal amounts of tissue or cell total protein (30 μg) were loaded on a 10% SDS/PAGE gel, transferred to a PVDF membrane (Merck Millipore), and detected using an ECL Western Blotting Detection System (Bio‐Rad). The information of the primary antibodies used in the current study was in accordance with the corresponding antibody information in the Star Methods in our previous publication.^7^

β‐Tubulin or glyceraldehyde 3‐phosphate dehydrogenase (GAPDH) was used as the loading control. Goat anti‐rabbit IgG‐HRP or goat anti‐mouse IgG‐HRP was used as secondary antibody. The gray intensity of the bands was quantified by ImageJ software.

### 2.6 Protein secondary structure prediction

The sequence of the first 200 (the number of predicated amino acids is limited in the algorithm) amino acids added to MET in ZM variant 4 (exon8 of *PTPRZ1* binding to exon2 of *MET*) was submitted to QUARK online service server (https://zhanglab.ccmb.med.umich.edu/QUARK2/). In the returned 3D structure, the additional segments to MET in the other three variants were highlighted in blue with PyMOL (2.3).

### 2.7 Data analysis and graphing

Survival analysis of patients was performed using GraphPad Prism 7 software. Log‐rank test was used to test the significance of difference of overall survival or progression‐free survival. Univariate and multivariate COX regression analyses were performed with SPSS 12.0. Statistical graph was obtained using SigmaPlot 14.0. In all statistical analysis, *p* value<0.05 was considered statistically significant.

## 3 RESULTS

### 3.1 A high‐sensitive and specific detection method of *ZM* fusions

The copy numbers of *ZM* fusions in different patients varies. As the driver role of *ZM* in glioma progression, it is necessary to detect its presence as early as we can.

**FIGURE 1:**
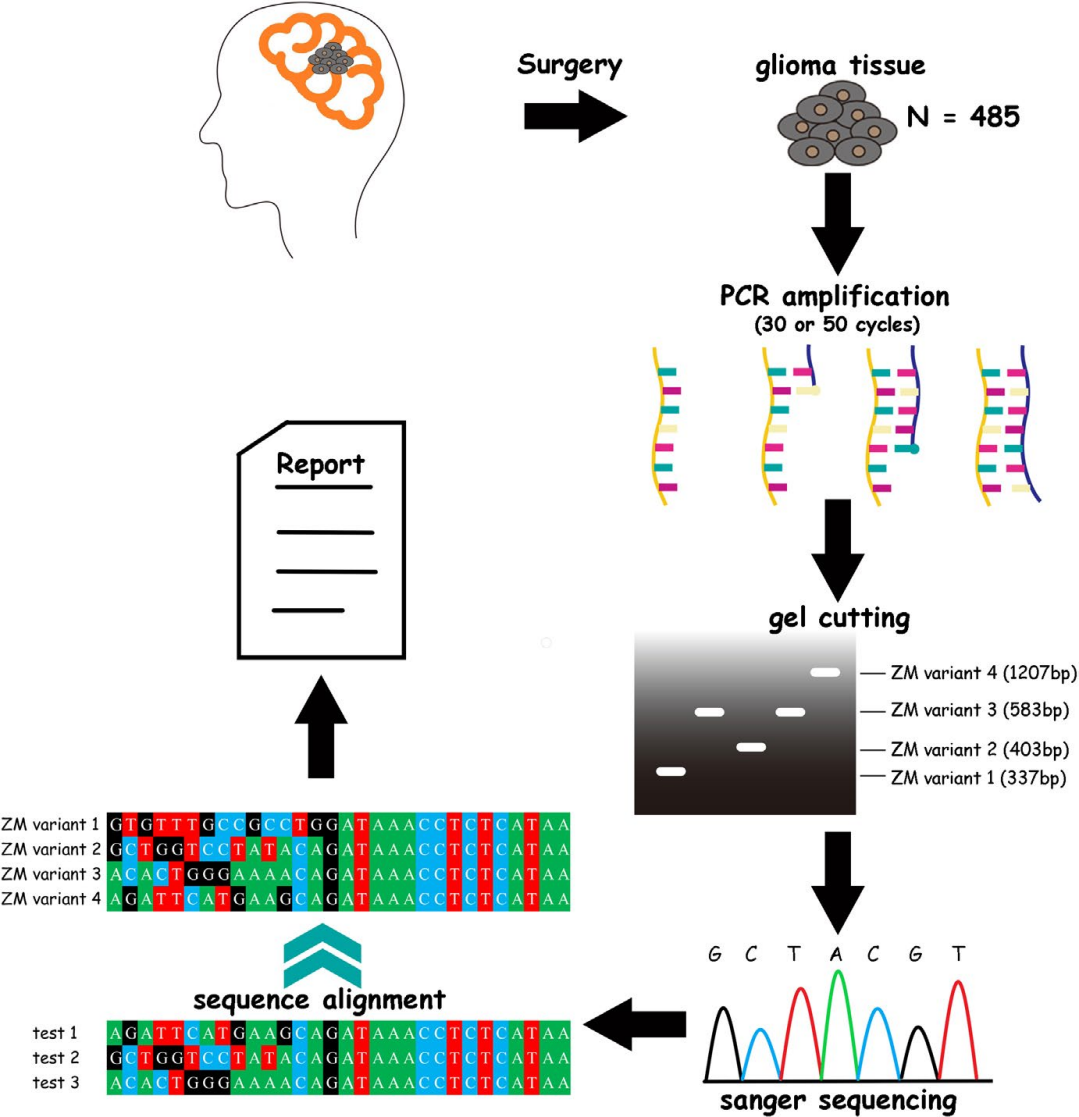
Illustration of the PCR‐sequencing diagnostic method. Glioma tissue RNA was abstracted after surgery. RT‐PCR of 50 amplification cycles then was performed with the primers designed based on the *PTPRZ1* and *MET* segments that are common to all the four ZM fusion variants. The PCR products of different *ZM* variants were of different sizes. All the PCR product bands on agarose gel were purified and sequenced via Sanger sequencing. The sequencing reads were aligned to the known *ZM* fusion sequences. The existence and the variant of ZM fusions then was confirmed and clinically reported.

We designed a method as performing two times of reverse transcriptase PCR (RT‐PCR) for each glioma tissue using primers flanking the fusion points. The numbers of amplification cycle in each RT‐PCR were separately 30 and 50. The PCR product bands in agarose gel were purified and sequenced using Sanger sequencing and the fusion point of *PTPRZ1* and *MET* were identified to confirm the presence of ZM fusions and which variant the fusion is (Figure 1). In every examination, a negative control test to avoid cross‐contamination or false positive and a positive control test to avoid false negative were performed simultaneously.

**FIGURE 2:**
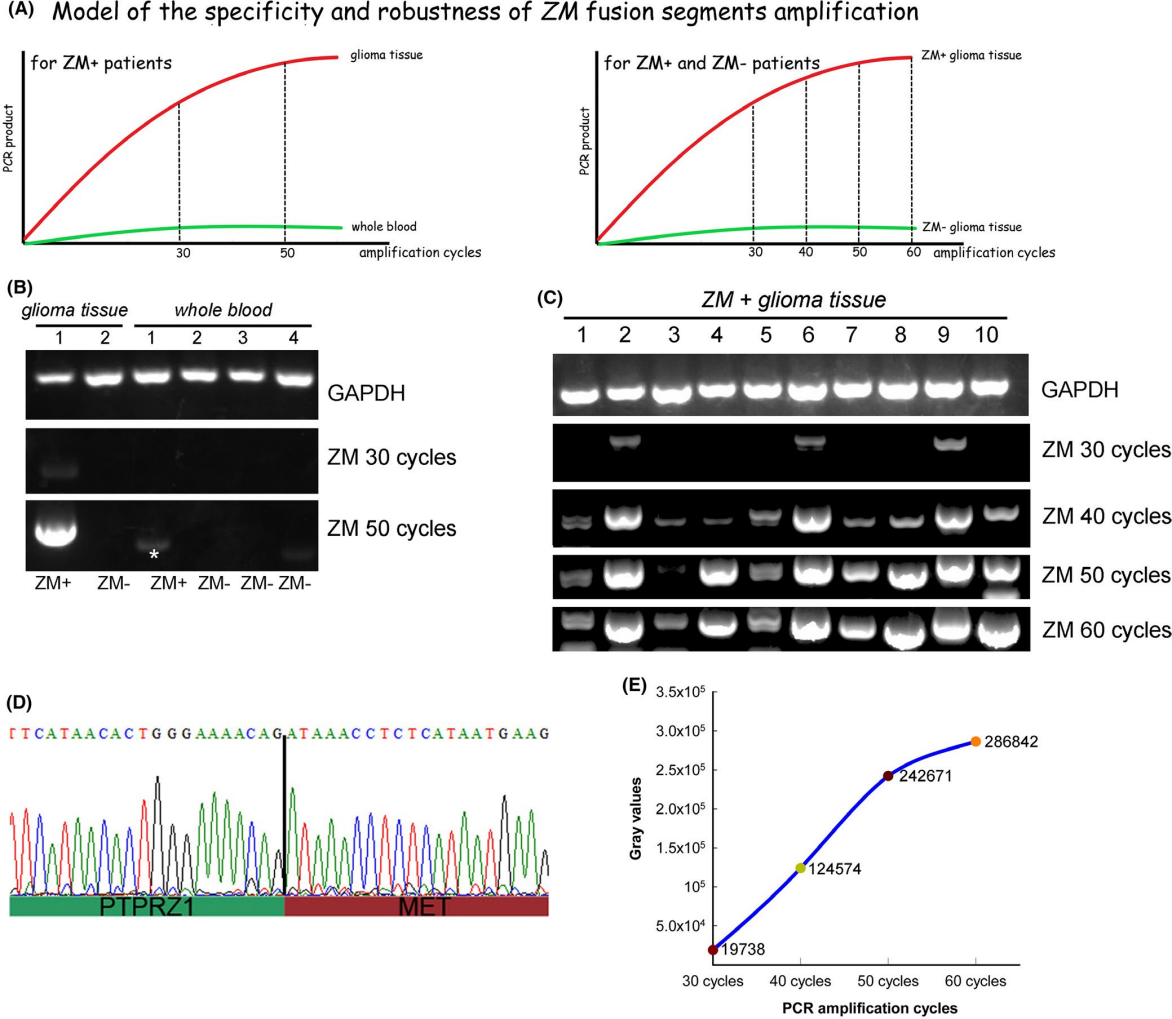
Demonstration of the specificity and robustness of *ZM* fusions segments amplification using reverse transcriptase PCR of 50 amplification cycles. A, Model of the ideal amplification method of *ZM* by RT‐PCR. The primers should be specific for *ZM* fusions and would not product any specific bands from control tissue such as the peripheral blood of the patients harboring *ZM*, or the glioma tissue from the patients without *ZM*. The quantity of *ZM* fragments should grow firmly along with increase in the number of amplification cycles. The number of amplification cycles at which the PCR products will not increase markedly anymore should be adopted as the amplification number used in an ideal detection method. B, The specificity of the amplification approach was evaluated through comparing *ZM*+ glioma tissue with *ZM*− glioma tissue, peripheral blood of the patients harboring *ZM*, and peripheral blood of the patients without *ZM* via RT‐PCR with 30 or 50 amplification cycles separately. A star indicates a non‐specific band of smaller size than the specific bands of *ZM* fusions. C, RT‐PCR amplifying *ZM* was separately performed with number of amplification cycles as 30, 40, 50, or 60. D, All the PCR product bands on agarose gel were purified and sequenced via Sanger sequencing and the fusion point of *PTPRZ1* and *MET* segment was confirmed. E, Growth curve of the sums of the gray intensity value of the PCR product bands derived from ten *ZM*+ glioma tissues at each cycle number in panel C.

In this method, RT‐PCR of 50 amplification cycles was employed so we could detect *ZM* fusions when they present with a small number of copies. The number of amplification cycles was much more than that commonly used in PCR. Thus, before we determined the mentioned *ZM* fusion detection method (PCR‐Sequencing), we firstly demonstrated the specificity and robustness of *ZM* fusions segments amplification using reverse transcriptase PCR of 50 amplification cycles (Figure 2). In the ideal amplification model, *ZM* fusion segment should be efficiently produced. Furthermore, as *ZM* fusions are somatic mutations, it should not be detected in *ZM*‐ glioma tissues or peripheral blood from *ZM*+ patients, even if the number of amplification cycles was large enough. (Figure 2A). To evaluate this method, we collected peripheral blood specimens from patients harboring *ZM* or not as control tissue. As expected, RNA extracted from *ZM*+ glioma tissue produced a remarkable band after RT‐PCR, whereas no specific PCR product could be detected with the whole peripheral blood RNA from *ZM*+ patient (Figure 2B).

Theoretically, quantity of specific PCR product grows exponentially along with numbers of amplification cycles until reaches the plateau phase. To detect early *ZM* fusions still present with fewer copies, we did a test to separately perform RT‐PCR with numbers of amplification cycles as 30, 40, 50, and 60 (Figure 2C). Among ten *ZM*+ glioma tissues, three tissues (samples noted as “2”, “6”, and “9”) could be identified *ZM*+ at 30 amplification cycles. Along with increase in amplification cycles numbers from 30 to 50, the quantity of PCR product grew. Until amplification cycle number reached 50, all the ten samples produced specific bands. Nevertheless, PCR product quantity kept stable and not increased markedly at 60 amplification cycles.

Before we finally certified the glioma samples as *ZM*+, all the PCR product bands on agarose gel were purified and sequenced via Sanger sequencing followed by confirmation of the fusion point of *PTPRZ1* and *MET* fragments (Figure 2D). The sums of the gray value of the PCR product bands from the ten *ZM*+ glioma tissues (Figure 2C) at each cycle number also indicated the trend of PCR product quantity along with amplification cycle numbers (Figure 2E). Considering that non‐specific amplification like primer‐dimer will also tend to occur after too much amplification, we proposed that 50 would be an ideal number of amplification cycle to detect *ZM* presenting with fewer copies and the subsequent Sanger sequencing, sequence alignment, and break point confirmation are necessary for a “gold standard” of *ZM*+ glioma diagnosis.

### 3.2 The incidence preference of *ZM* fusions

**FIGURE 3:**
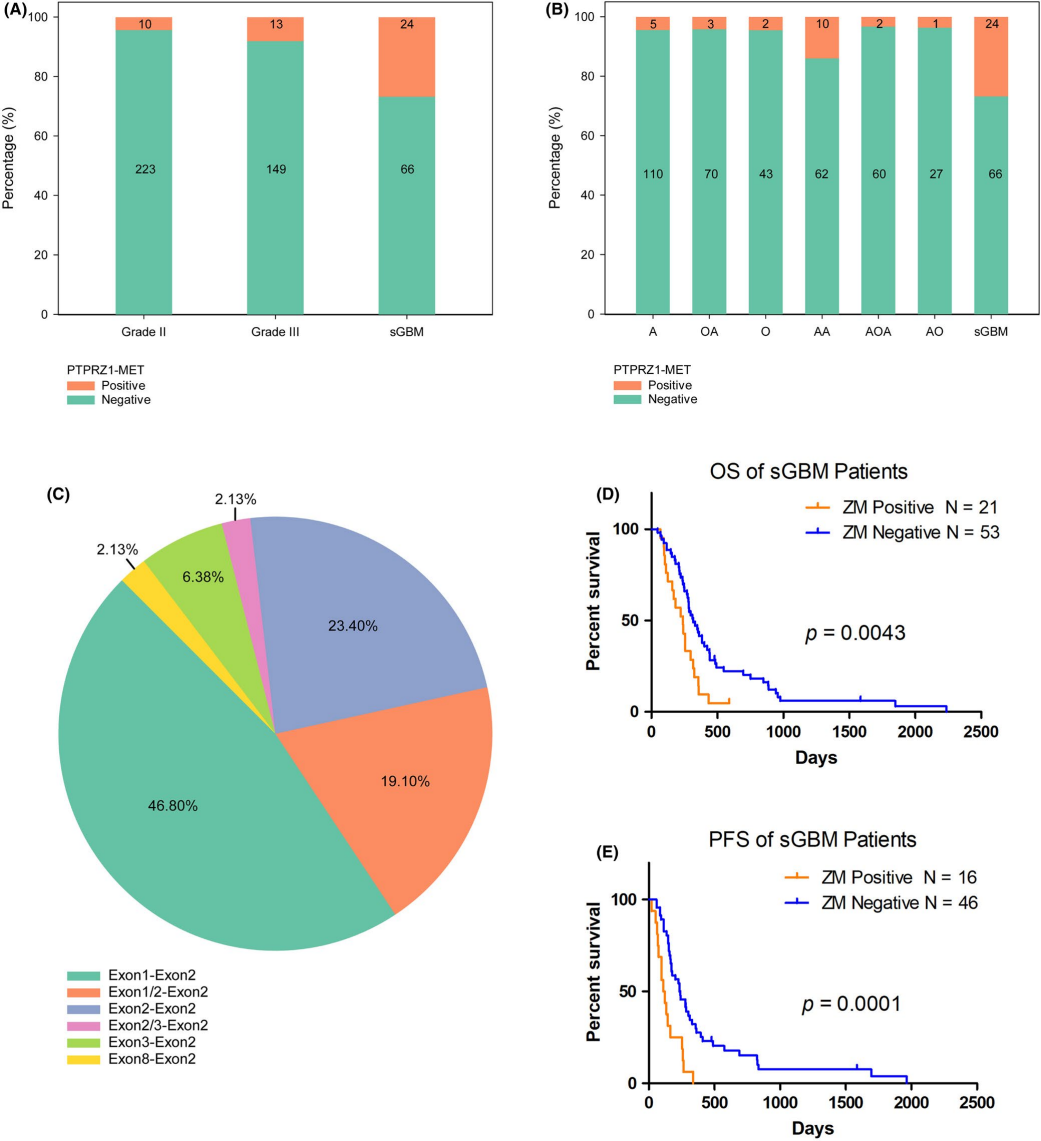
Distribution characteristics of *ZM* variants. A, Incidence of *ZM* fusions in gliomas of different WHO grades. B, Incidence of *ZM* fusions in histological subgroups of glioma (A: astrocytoma, grade II; OA: oligoastrocytomas, grade II; O: oligodendroglioma, grade II; AA: anaplastic astrocytoma, grade III; AOA: anaplastic oligoastrocytomas, grade III; AO: anaplastic oligodendroglioma, grade III). C, Proportion of the four *ZM* fusion variants. The percentages show the relative frequencies of each *ZM* fusion variant in all *ZM* fusions identified in this study. D, Kaplan‐Meier curve of overall survival (OS) for patients with sGBM with (N = 21) or without (N = 53) *ZM* fusion. E, Kaplan‐Meier curve of progression‐free survival (PFS) for patients with sGBM with (N = 16) or without (N = 46) *ZM* fusion.

Using the above‐stated diagnosis approach, we examined the incidence of *ZM* fusions in a cohort consisting of 485 adults with WHO grade II–IV diffuse glioma from different regions in China. *ZM* fusions occurred more frequently in sGBMs than in grade II and grade III gliomas (Figure 3A, Pearson chi‐squared test, χ^2^ = 33.657, *p* < 0.001). As a contrast, none of the primary GBM was found *ZM*+, which was in accordance with our previous discovery that *ZM* was a driver of the progression from LGG to sGBM. *ZM* fusions were more frequent in grade III astrocytomas (AA, 13.9%) and sGBMs (26.7%), whereas only 3.2% of the grade III oligoastrocytomas (AOA) harbored *ZM* fusions (Figure 3B) suggesting a preference in gliomas with astrocyte origin. The 24 sGBM samples harboring *ZM* fusions include 4 *ZM*+ samples involved in our previous study.^7^ All other *ZM* fusions positive specimens were firstly reported here.

Four *ZM* fusion variants involving four different breakpoints: exon 1, 2, 3, or 8 within *PTPRZ1* coding sequence and one common breakpoint (exon 2) within *MET* were confirmed. Fusions with exon 1 or 2 of *PTPRZ1* fused to exon 2 of *MET* (Exon1‐Exon2 or Exon2‐Exon2, respectively) were the most common variants (46.8% and 23.4%, respectively) (Figure 3C). Three of the four variants we found and confirmed through the proposed RT‐PCR method, the *ZM* variant 1, 2, and 4 separately with exon 1, 2, or 8 of *PTPRZ1* fused to exon 2 of *MET* had been previously reported in pediatric glioblastoma.^10^ *ZM* variant 3 with exon 3 of *PTPRZ1* fused to exon 2 of *MET* was our unique discovery and had not been previously reported.

Based on diagnosis results given by RT‐PCR examination, we confirmed that *ZM* conferred a negative survival effect in sGBM patients. Kaplan‐Meier survival analysis demonstrated poor overall survival (OS) for patients with *ZM* fusion‐positive sGBM compared with sGBM patients without *ZM* fusion (median OS: 239 d vs. 318 d, respectively; *p* = 0.0043, log‐rank test) (Figure 3D, Table S1). A similar trend was found for progression‐free survival (PFS) (median PFS: 116 d vs. 240 d, respectively; *p* = 0.0001, log‐rank test) (Figure 3E, Table S1). In Univariate and Multivariate Cox regression analyses, *ZM* fusions exhibited independent prognostic value for glioma patients (Table 1).

**TABLE 1:** Univariate and multivariate analysis of OS in sGBM patients. Univariate and Multivariate Cox regression analyses of ZM fusions and several other clinical variables in CGGA.

Variables	Univariate analysis		Multivariate analysis	
	HR (95% CI)	*p* value	HR (95% CI)	*p* value
ZM fusion	1.663 (1.095–2.526)	0.017	23.039 (4.637–114.479)	<0.001
Age at Diagnosis	0.711 (0.507–0.997)	0.048	1.011 (0.964–1.059)	0.664
Gender	0.802 (0.562–1.143)	0.222	—	—
IDH1 mutation status	0.656 (0.424–1.016)	0.059	0.244 (0.083–0.715)	0.010
MGMT methylation	1.159 (0.638–2.105)	0.627	2.369 (1.032–5.436)	0.042
1p19q co‐deletion	0.577 (0.282–1.184)	0.134	0.616 (0.185–2.052)	0.430
Chemotherapy	0.833 (0.583–1.188)	0.313	—	—

**FIGURE 4:**
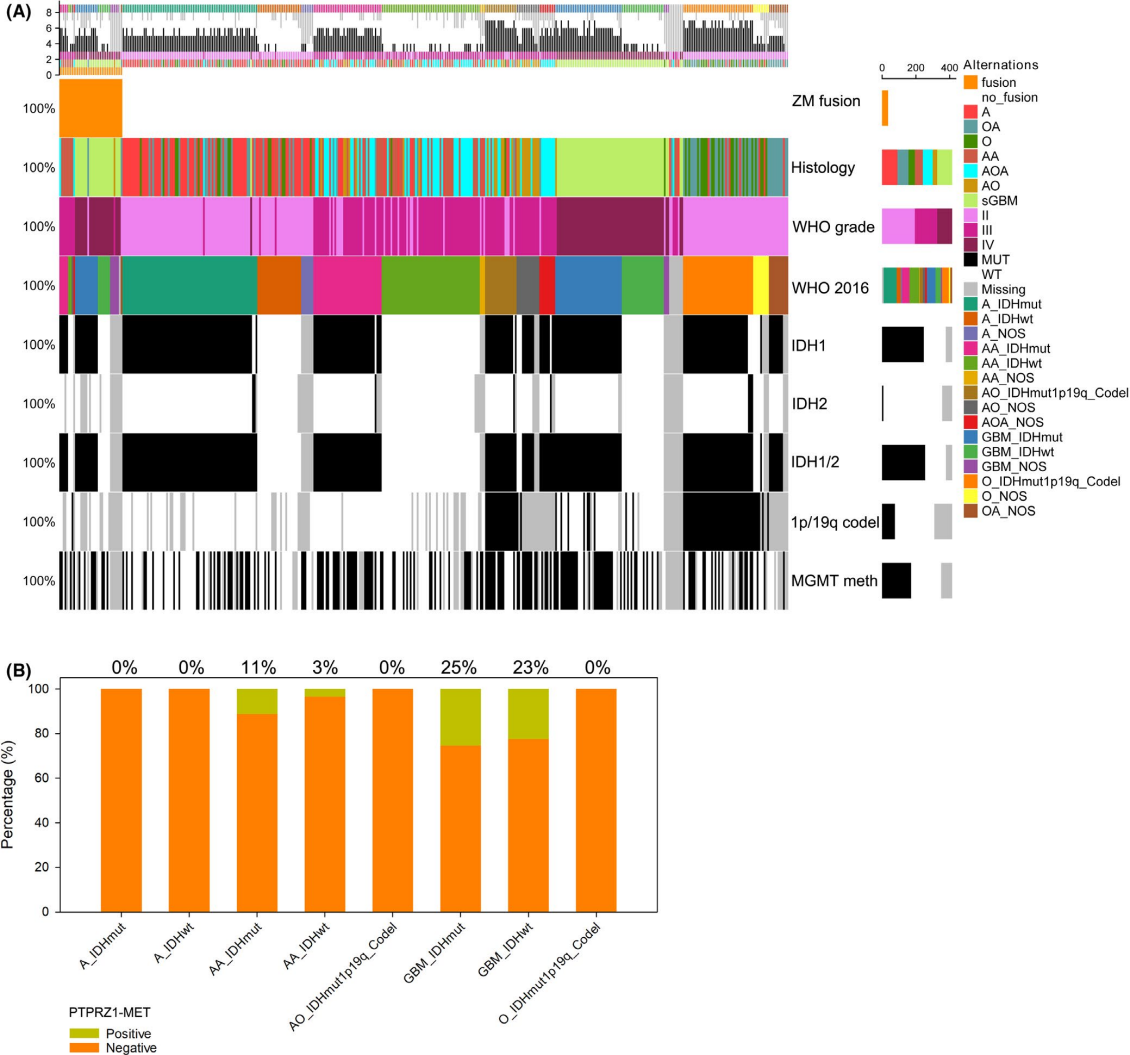
The correlations of *ZM* fusion incidence with the 2016 WHO classification of glioma. A, The landscape of *ZM* fusions and the critical histology and molecular features counted in the definition of 2016 WHO classification of glioma. B, Distribution of *ZM* fusions in tumors with 2016 WHO classification of glioma. (the specimens do not fit into any narrowly defined classifications which were labeled as “NOS” were not counted). NOS: not otherwise specified.

The landscape of *ZM* fusions and the critical histology and molecular features counted in the definition of 2016 WHO classification of glioma^2^ revealed the correlations of *ZM* fusion incidence with the 2016 WHO classification of glioma (Figure 4A). *ZM* fusions distributed exclusively in AA and GBM, whereas their distribution did not show specific preference depending *IDH* status (Figure 4B, the specimens do not fit into any narrowly defined classifications which were labeled as “NOS” were not counted).

**FIGURE 5:**
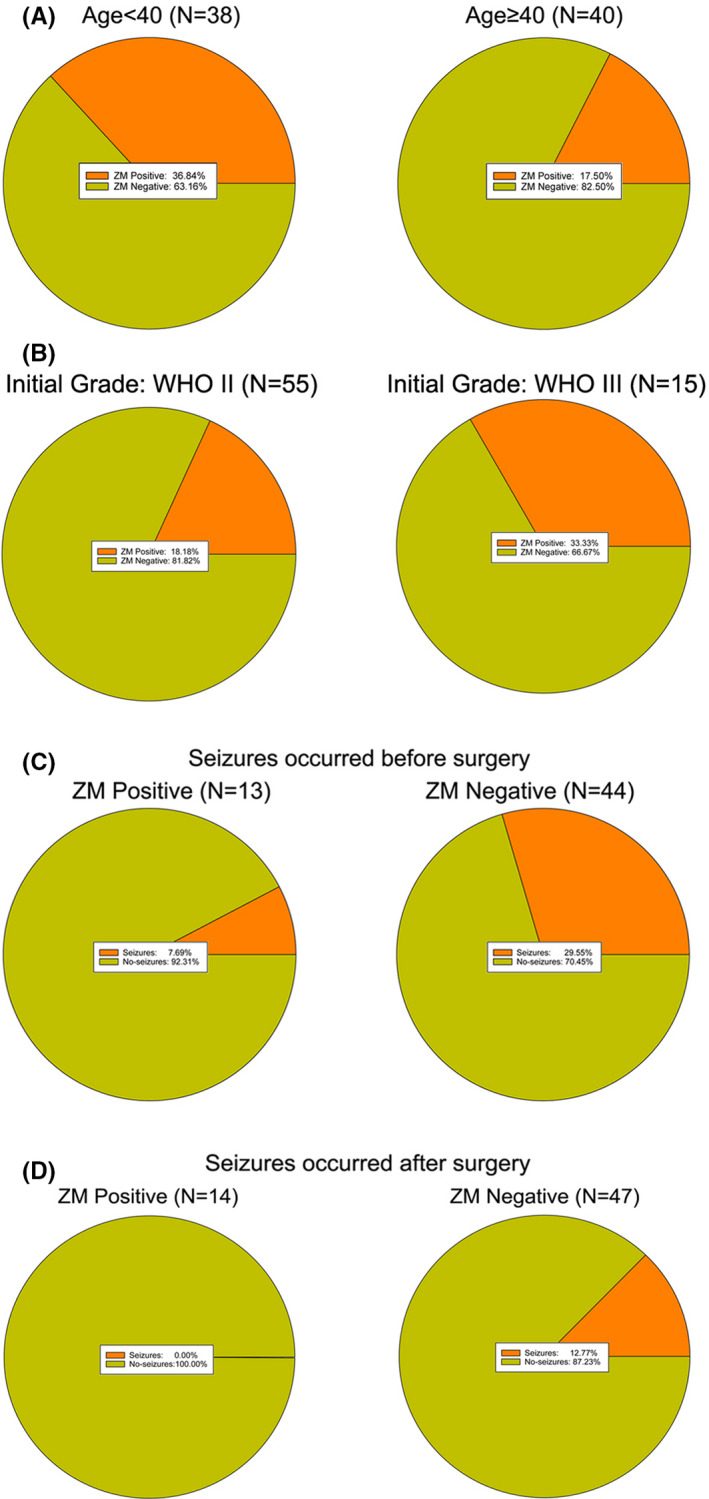
The clinical features of sGBM patients harboring *ZM* fusion. A, The incidence of *ZM* fusions in younger (Age <40) or older (Age ≥40) patients. B, The incidence of *ZM* fusions in patients with sGBM progressed from LGG of WHO grade II or III. C, The occurrence rates of seizure in patients with sGBM harboring *ZM* fusions or not before surgical resection. D, The occurrence rates of seizure in patients with sGBM harboring *ZM* fusions or not after surgical resection.

We further investigated the clinical features of sGBM patients harboring *ZM* fusion or not. The incidence of *ZM* fusion in younger patients (Age < 40) was higher (36.84% vs. 17.50%) than elder (Age ≥ 40) patients (Figure 5A, Table S2). In the cohort of 55 patients with sGBM progressed from LGGs of WHO II, *ZM* fusion incidence was 18.18%, whereas in patients who had been initially diagnosed with glioma of WHO III grade before sGBM (*N* = 15), the incidence of *ZM* fusion was 33.33% (Figure 5B, Table S2). As epileptic seizure is a component of syndrome of LGG and glioblastoma, we explored the correlation between the occurrence of seizures and *ZM* fusion incidence. Before surgical resection, the occurrence rate of seizures in patients with *ZM*+ sGBM was 7.69% (Figure 5C, Table S2); significantly lower than the occurrence rate of seizures in patients with *ZM*− sGBM (29.55%). After surgical resection, the occurrence rate of seizures in patients with *ZM*‐ sGBM was prominently reduced to 12.77%. Meanwhile, none of the patients with *ZM*+ sGBM had seizures occurred after surgical resection (Figure 5D, Table S2).

### 3.3 The coiled‐coil structures in ZM fusions contribute to ligand‐independent MET activation

**FIGURE 6:**
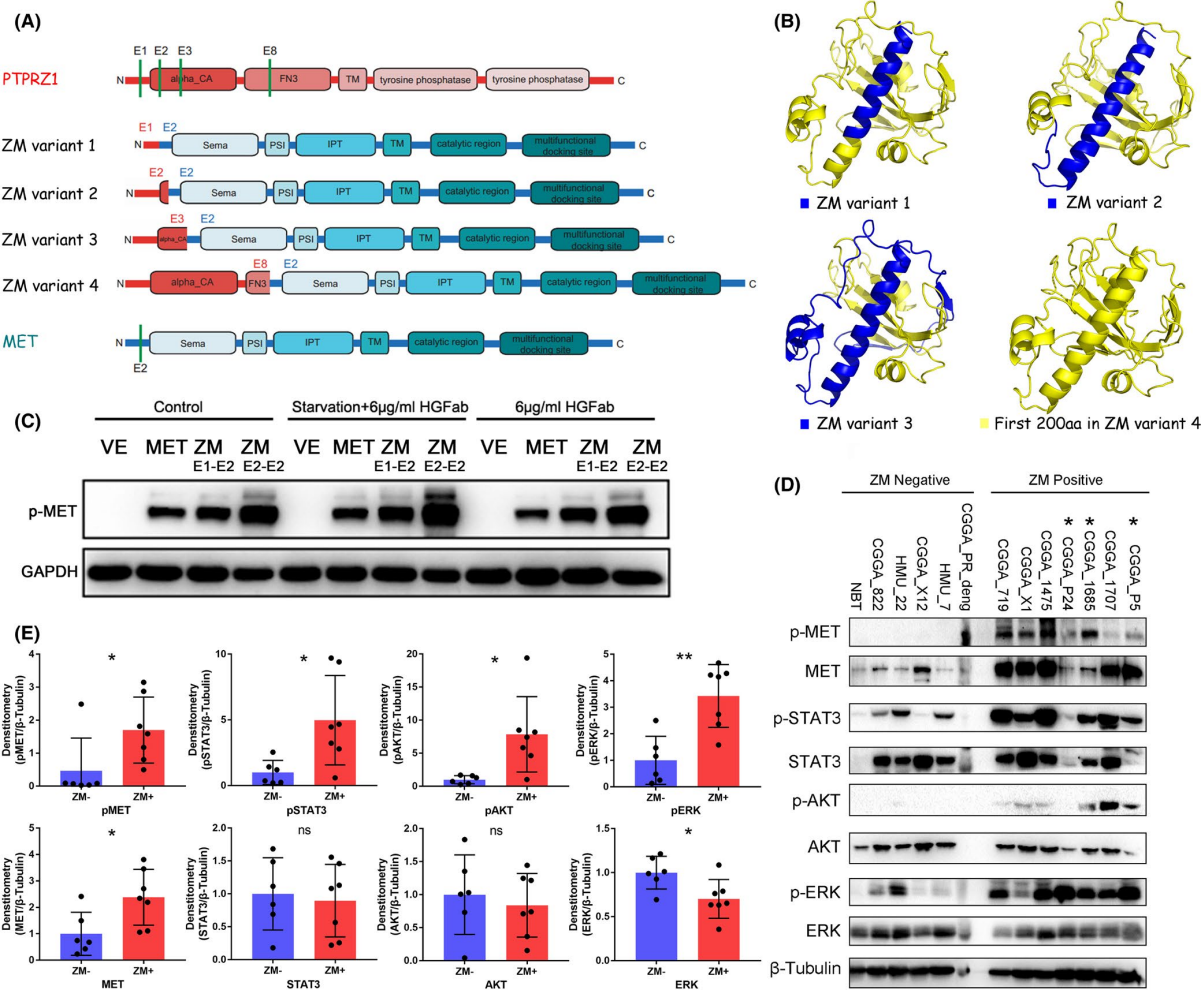
Coiled‐coil structures in ZM contribute to ligand‐independent MET activation. A, Diagram that MET is modified by PTPRZ1 fragment adding to SEMA domain. B, The 3D protein structure predicated using QUARK algorithm. The predicated structure of the first 200 amino acids added to MET in ZM variant 4 (exon8 of PTPRZ1 binding to exon2 of MET) is shown in yellow, and the PTPRZ1 segments in ZM variant 1, 2, and 3 are separately highlighted in blue. C, *ZM* fusion leads to MET hyperactivation in a ligand‐independent manner. Following 12 h of serum starvation and HGF blocking with 6 μg/ml of HGF antibody, MET hyperactivation caused by adenoviral vector‐mediated transient expression of *ZM* fusion (variant 1 or 2) in U87 MG cells was not impaired. VE: vector control. D, Immunoblottings show strong phosphorylation of MET and its down‐stream signaling in *ZM* fusion‐positive glioma compared with normal brain tissue (NBT) and *ZM*‐free glioma. CGGA_P24, CGGA_1685, and CGGA_P5 were diagnosed as *ZM* fusion positive via the detection method with 50 amplification cycles (indicated by asterisk). HMU: ID for patients from Harbin Medical University. E, The gray intensity of the immunoblotting bands in (D). *p < 0.05, **p < 0.01, ns: no statistically significant.

In previous studies, we had revealed that the abnormal activation of MET signaling caused by *ZM* fusions was a critical mechanism of driving LGGs progress to sGBM.^7^ To further illustrate the mechanism of MET hyperactivation in gliomas harboring *ZM* fusions, we analyzed the protein structure of the partner fragment of MET (the PTPRZ1 fragment) in PTPRZ1‐MET. Via QUARK, an algorithm determining spatial location of every atom in a protein molecule from the amino acid sequence developed by Dong Xu and Yang Zhang.^11^, ^12^ We predicted the 3D structure of the first 200 amino acids (the number of predicated amino acids is limited in the algorithm) added to MET in ZM variant 4 (exon8 of *PTPRZ1* binding to exon2 of *MET*). As ZM variant 4 is the longest PTPRZ1‐MET fusion, the additional fragments of PTPRZ1 in all the other three ZM variants are included in these 200 amino acids (Figure 6A). The additional segments to MET in all these four variants contained remarkable coiled‐coil motifs (Figure 6B, Figure S1). These characteristic structure of coiled‐coil often indicating ligand‐independent dimerization and kinase hyperactivation.^13^, ^14^

To verify whether the ZM fusions could cause a ligand‐independent kinase hyperactivation or not. Experiments *in vitro* were performed in U87 MG cell line with adenoviral‐mediated expression of *ZM* variant 1 or 2. The results showed that MET signaling activation remained persistent following blockage of HGF with anti‐HGF antibodies in FBS‐free medium (Figure 6C), suggesting *ZM* fusions caused MET hyperactivation in a ligand‐independent manner. We examined MET signaling activity in seven *ZM*+ glioma tissue samples, five *ZM*− gliomas, and a normal brain tissue. Immunoblotting analyses showed strong phosphorylation of MET and its down‐stream signaling including STAT3, ERK, and AKT in *ZM*+ tumors (Figure 6D, E). Among the *ZM*+ tumors, CGGA_P24, CGGA_1685, and CGGA_P5 were diagnosed as *ZM* fusion positive via the detection method with 50 amplification cycles as above mentioned (Figure 2).

## 4 DISCUSSION

We had previously reported the presence of *ZM* fusion in glioma.^15^ Subsequently, we elaborated that *ZM* fusions co‐occurred with *MET*‐exon‐14‐skipping, and both two *MET* alterations promoted glioma progression. Based on these findings, patients with *ZM*+ high‐grade glioma (WHO III and IV) had been enrolled in a clinical trial of blocking malignant progression with a highly selective and BBB‐permeable MET kinase inhibitors.^7^

In tumors, the canonical driver genetic alterations could be acquired and seeded very early.^16^ Since the linear progression model of cancers indicating that cancer progression is closely depended on accumulation of somatic alterations is one of the most widely accepted theory,^17^, ^18^ heterogeneity of critical alterations and the resulted distinct subpopulation of cancer cells have been established in early primary tumor.^19^, ^20^ Considering that *ZM* fusions are critical drivers of glioma progression and the clonal evolution of *ZM* fusion copies, detection of these fusions even if they are present in glioma tissues with few copy numbers is meaningful and will benefit the patients.

However, the feature of fusion genes including two sequences from two totally different genes decides the difficulties of their detection, especially in clinical diagnosis. The antibodies for either of the two fusion partners are not specific enough for the fusion protein, while development of a specialized antibody targeting the fusion point is arduous. Fluorescent in situ hybridization (FISH) has a tedious operating process and the diagnostic results prone to be bias depending on the field of the pathological slices under the microscope. It means that FISH is not an ideal approach to detect *ZM* fusions as this early seeding genetic alterations might be dispersive in tissue bulk with very fewer copies. Bulk tumor with gene rearrangements might be missed as negative via FISH examination while be identified as positive by qRT‐PCR analysis.^21^ Besides, the “off‐target” hybridization is common because of the high‐level of sensitivity of FISH.^22^ This defect might exaggerate the incidence of the genomic alterations.

Real‐time PCR is a commonly used method to detect clinically meaningful genetic alterations in cancers. However, real‐time PCR is not applicable to detect ZM fusions. The quantify capacity of real‐time PCR assay is based on the stable amplification efficiency of primers. Whereas, ZM fusions contain four variants of different lengths. The amplification efficiency of the primers is difficult to keep consistent when different ZM fusion variants are detected. For real‐time PCR, the optimal PCR product size is between 100 and 150 base pairs (https://www.sigmaaldrich.com/content/dam/sigma‐aldrich/docs/Sigma/Bulletin/qr0100bul.pdf, Technical bulletin of quantitative RT‐PCR kit from Sigma‐Aldrich). PCR product of such length is not applicable for gel cutting from agarose gel after electrophoresis and the following PCR product purification. As Sanger sequencing and sequence alignment with the known *ZM* fusion gene sequences is required for ZM detection, the procedure of PCR product purification is needed.

In the current study, we proposed a detection approach containing two times of reverse transcriptase PCR (RT‐PCR) with 30 or 50 amplification cycles separately to amplify *ZM* fusion segments before Sanger sequencing. We proved that the relative larger number of amplification cycles could ensure clearly confirmation of *ZM* fusions with fewer copies. The subsequent Sanger sequencing of the PCR products and sequence alignment with the known *ZM* fusion gene sequences would make the fusion point of *PTPRZ1* and *MET* visualized and further confirm the presence of *ZM* fusions. Moreover, the RT‐PCT and Sanger sequencing techniques are low cost.

With the proposed PCR‐Sequencing approach, we described the incidence features of *ZM* fusion genes. The incidence was found higher than our previous reports, in which *ZM* fusions were detected by RNA‐seq and confirmed by RT‐PCR.^7^ As the repressive effects of MET inhibitor in malignant progression of *ZM*+ glioma had emerged, the early detection of these alterations will benefit the patients in prevention or early management.

MET signaling activation is dependent on binding of MET kinase with its ligand HGF.^23^, ^24^ The large N‐terminal domain of MET (the Sema domain) is necessary for HGF binding and dimerization.^25^, ^26^, ^27^ In ZM fusion protein, the MET structure remained intact overall, whereas the Sema domain was modified by PTPRZ1 fragment,^15^ which might be the cause of ligand‐independent phosphorylation of the MET kinase domain.

In classic model of receptor tyrosine kinase (RTK) activation, ligand binds to the extracellular ligand pocket and causes two monomers’ combination in a process termed as dimerization.^28^ Such the active signaling brought by ligand would be transduced to intracellular domain. Subsequently, the intracellular kinase domain is auto‐phosphorylated, and the growth receptor becomes catalytically active.^28^, ^29^

The dimerization mechanisms vary in different RTKs. The main mechanisms are the three ones as following: Each RTK monomer binds to a ligand molecule and then dimerizes; two monomers are linked by a single ligand molecule and then dimerizes; or two monomers are originally linked by a disulfide bond in their extracellular region, and after ligand binding, the intracellular domains conformation change and bind each other.^29^, ^30^

For MET, the extracellular SEMA domain is the key structure for ligand binding and dimerization. In ZM fusions, the fusion partner sections are added to the SEMA domain, suggesting that the modification prone to affect the critical step of ligand binding and dimerization of MET kinase. To verify this hypothesis, we performed *in vitro* experiments with U87 MG cell line. The results suggested that *ZM* fusions could cause MET hyperactivation in a ligand‐independent manner. As a primary GBM cell line, the use of U87 MG had potential limitation to study the malignant progression from LGG to sGBM. However, considering the lack of low‐grade or secondary glioma cell lines, U87 MG cell line was still a reasonable and reliable choice. Besides, as a MET‐dependent glioma cell line,^31^ U87 MG was widely used in MET signaling pathway‐related researches.^32^, ^33^

The incidence of *ZM* fusions in different age of patients with sGBM was distinct. The occurrence rate of seizures was also different in patients depending on whether their tumors harboring *ZM* fusions or not. This finding suggested that the *ZM* fusion status may serve as a potential indicator of epileptic seizures and surgical seizure and the detection of *ZM* fusion could provide a more accurate assessment of personalized treatment in clinic. The above clinical relevance suggested that there is still a long way before fully uncovering the influence of the existence of *ZM* fusions in glioma progression.

## CONFLICT OF INTEREST

The authors declare that they have no conflict of interests.

## CONSENT TO PARTICIPATE

Informed consent was obtained from all individual participants for CGGA project in this study.

## Supporting information

Supplementary Material

## Data Availability

The data that support the findings of this study are available from the corresponding author upon reasonable request.
