# Supplementary material for: High‐sensitive clinical diagnostic method for PTPRZ1‐MET and the characteristic protein structure contributing to ligand‐independent MET activation
Source: CNS Neurosci Ther. 2021 Feb 28;27(5):617–28. doi: 10.1111/cns.13627 (PMC8025647; doi:10.1111/cns.13627)
Supplement: Supplementary file 1 — Supplementary Material [file CNS-27-617-s001.pdf]

Supplementary Figure 1

>Exon 1- Exon 2
MRILKRFLACIQLLCVCRLDKPLI

> Exon 2- Exon 2
MRILKRFLACIQLLCVCRLDWANGYYRQQRKLVEEIGWSYTDKPLI

> Exon 3- Exon 2
MRILKRFLACIQLLCVCRLDWANGYYRQQRKLVEEIGWSYTGALNQKNWGKKYPTCNSPK
QSPINIDEDLTQVNVNLKKLKFQGWDKTSLENTFIHNTGKTDKPLI

> Exon 8- Exon 2
MRILKRFLACIQLLCVCRLDWANGYYRQQRKLVEEIGWSYTGALNQKNWGKKYPTCNSPK
QSPINIDEDLTQVNVNLKKLKFQGWDKTSLENTFIHNTGKTVEINLTNDYRVSGGVSEMV
FKASKITFHWGKCNMSSDGSEHSLEGQKFPLEMQIYCFDADRFSSFEEAVKGKGKLRALS
ILFEVGTEENLDFKAIIDGVESVSRFGKQAALDPFILLNLLPNSTDKYYIYNGSLTSPPC
TDTVDWIVFKDTVSISESQLAVFCEVLTMQQSGYVMLMDYLQNNFREQQYKFSRQVFSSY
TGKEEIHEADKPLI

Supplementary Table 1

SampleID	OS（day）	Censor（OS）	PFS （day）	Censor (PFS)	ZM Fusion Variant
CGGA_1748	46	1	NA	1	no_fusion
CGGA_1068	68	1	68	1	2-2
CGGA_1726	69	1	NA	1	no_fusion
CGGA_1396	75	1	NA	1	no_fusion
CGGA_1375	92	1	92	1	no_fusion
CGGA_1397	93	1	74	1	1-2
CGGA_1532	96	1	96	1	2-2
CGGA_719	101	1	51	1	8-2
CGGA_1676	111	1	111	1	2-2
CGGA_181	114	1	114	1	no_fusion
CGGA_1637	114	1	NA	1	no_fusion
CGGA_1272	124	1	63	1	2-2
CGGA_D34	147	1	86	1	no_fusion
CGGA_1230	154	1	NA	1	no_fusion
CGGA_P2	158	1	97	1	2-2
CGGA_1129	168	1	NA	1	1-2
CGGA_1609	175	1	173	1	no_fusion
CGGA_1752	181	1	59	1	no_fusion
CGGA_1475	182	1	20	1	2/3-2
CGGA_1199	206	1	114	1	no_fusion
CGGA_1460	209	1	152	1	no_fusion
CGGA_518	212	1	59	1	no_fusion
CGGA_P1	220	1	NA	NA	2-2
CGGA_1519	221	1	NA	1	no_fusion
CGGA_1136	236	1	NA	1	no_fusion
CGGA_272	238	1	114	1	no_fusion
CGGA_1707	239	1	NA	1	1-2
CGGA_1730	242	1	121	1	1-2
CGGA_1573	248	1	148	1	no_fusion
CGGA_1296	249	1	166	1	no_fusion
CGGA_1188	256	1	134	1	2-2
CGGA_1683	256	1	164	1	1-2
CGGA_P9	270	1	157	1	no_fusion
CGGA_1393	275	1	233	1	no_fusion
CGGA_1490	280	1	NA	1	no_fusion
CGGA_1602	284	1	148	1	no_fusion
CGGA_1346	284	1	240	1	no_fusion
CGGA_1218	286	1	164	1	no_fusion
CGGA_X1	296	1	145	1	2-2
CGGA_1412	296	1	280	1	no_fusion
CGGA_226	310	1	202	1	no_fusion
CGGA_X4	316	1	254	1	1-2/2-2

CGGA_220	318	1	226	1	no_fusion
CGGA_1733	324	1	263	1	1-2
CGGA_1495	330	1	85	1	no_fusion
CGGA_1170	346	1	285	1	no_fusion
CGGA_1628	354	1	232	1	no_fusion
CGGA_P6	356	1	NA	NA	1-2
CGGA_1405	357	1	257	1	1-2/2-2
CGGA_462	361	1	136	1	no_fusion
CGGA_X7	383	1	NA	NA	no_fusion
CGGA_1506	384	1	172	1	no_fusion
CGGA_1298	400	1	280	1	no_fusion
CGGA_1766	422	1	362	1	no_fusion
CGGA_1711	432	1	NA	1	1-2/2-2
CGGA_1665	440	1	356	1	no_fusion
CGGA_1175	441	1	330	1	no_fusion
CGGA_773	443	1	153	1	no_fusion
CGGA_X6	478	0	478	0	no_fusion
CGGA_1622	484	1	241	1	no_fusion
CGGA_374	493	1	409	1	no_fusion
CGGA_1751	548	1	301	1	no_fusion
CGGA_X2	590	0	338	1	1-2/2-2
CGGA_1625	696	1	178	1	no_fusion
CGGA_1585	751	1	690	1	no_fusion
CGGA_1324	848	1	490	1	no_fusion
CGGA_1366	885	1	824	1	no_fusion
CGGA_1632	888	1	827	1	no_fusion
CGGA_1197	942	1	395	1	no_fusion
CGGA_1450	957	1	835	1	no_fusion
CGGA_1652	976	1	576	1	no_fusion
CGGA_1734	1585	0	1585	0	no_fusion
CGGA_1547	1849	1	1695	1	no_fusion
CGGA_822	2237	1	1964	1	no_fusion
CGGA_1186	NA	1	NA	1	no_fusion
CGGA_109	NA	1	281	0	no_fusion
CGGA_1105	NA	0	313	1	no_fusion
CGGA_D59	NA	NA	NA	NA	no_fusion
CGGA_899	#N/A	#N/A	#N/A	#N/A	no_fusion
CGGA_1285	#N/A	#N/A	#N/A	#N/A	no_fusion
CGGA_1283	#N/A	#N/A	#N/A	#N/A	no_fusion
CGGA_1099	#N/A	#N/A	#N/A	#N/A	no_fusion
CGGA_X3	#N/A	#N/A	#N/A	#N/A	1-2/2-2
CGGA_1271	#N/A	#N/A	#N/A	#N/A	no_fusion
CGGA_632	#N/A	#N/A	#N/A	#N/A	no_fusion

CGGA_1370	#N/A	#N/A	#N/A	#N/A	no_fusion

Supplementary Table 2

SampleID	Age(≥40)	ZM Fusion Variant	Initial grade (WHO)	Seizures before surgery	Seizures after surgery
CGGA_1397	0	1-2	3	No	No
CGGA_1129	0	1-2	2	No	No
CGGA_1707	0	1-2	3	No	No
CGGA_1733	0	1-2	2	#N/A	#N/A
CGGA_X4	0	1-2/2-2	#N/A	#N/A	No
CGGA_1405	0	1-2/2-2	#N/A	#N/A	#N/A
CGGA_X2	0	1-2/2-2	#N/A	#N/A	#N/A
CGGA_1475	0	2/3-2	3	No	No
CGGA_1068	0	2-2	2	No	No
CGGA_1676	0	2-2	3	Yes	No
CGGA_1272	0	2-2	3	No	No
CGGA_P2	0	2-2	2	#N/A	#N/A
CGGA_1188	0	2-2	2	No	No
CGGA_X1	0	2-2	#N/A	#N/A	#N/A
CGGA_1748	0	no_fusion	3	#N/A	No
CGGA_1726	0	no_fusion	2	No	No
CGGA_181	0	no_fusion	2	No	No
CGGA_1752	0	no_fusion	2	#N/A	#N/A
CGGA_1199	0	no_fusion	2	Yes	No
CGGA_518	0	no_fusion	2	Yes	No
CGGA_1136	0	no_fusion	3	No	No
CGGA_1296	0	no_fusion	2	No	No
CGGA_220	0	no_fusion	2	No	No
CGGA_1170	0	no_fusion	3	No	No
CGGA_462	0	no_fusion	2	Yes	Yes
CGGA_1506	0	no_fusion	2	Yes	No
CGGA_1298	0	no_fusion	2	No	Yes
CGGA_1665	0	no_fusion	2	No	No
CGGA_1175	0	no_fusion	2	Yes	No
CGGA_374	0	no_fusion	2	#N/A	#N/A
CGGA_1751	0	no_fusion	2	#N/A	No
CGGA_1632	0	no_fusion	2	No	#N/A
CGGA_1450	0	no_fusion	2	Yes	Yes
CGGA_1652	0	no_fusion	2	No	Yes
CGGA_1547	0	no_fusion	2	Yes	Yes
CGGA_109	0	no_fusion	2	No	No
CGGA_1105	0	no_fusion	2	Yes	No
CGGA_D59	0	no_fusion	2	#N/A	#N/A
CGGA_1730	1	1-2	2	No	No
CGGA_1683	1	1-2	#N/A	No	No
CGGA_P6	1	1-2	2	#N/A	#N/A

CGGA_1711	1	1-2/2-2	#N/A	No	No
CGGA_1532	1	2-2	2	No	No
CGGA_P1	1	2-2	2	#N/A	#N/A
CGGA_719	1	8-2	2	No	No
CGGA_1396	1	no_fusion	3	No	No
CGGA_1375	1	no_fusion	2	No	No
CGGA_1637	1	no_fusion	3	No	No
CGGA_D34	1	no_fusion	2	#N/A	#N/A
CGGA_1230	1	no_fusion	2	No	No
CGGA_1609	1	no_fusion	2	No	No
CGGA_1460	1	no_fusion	3	No	No
CGGA_1519	1	no_fusion	2	No	No
CGGA_272	1	no_fusion	2	No	No
CGGA_1573	1	no_fusion	2	Yes	Yes
CGGA_P9	1	no_fusion	2	#N/A	No
CGGA_1393	1	no_fusion	2	Yes	No
CGGA_1490	1	no_fusion	2	No	No
CGGA_1602	1	no_fusion	2	No	No
CGGA_1346	1	no_fusion	2	No	No
CGGA_1218	1	no_fusion	2	No	No
CGGA_1412	1	no_fusion	2	No	No
CGGA_226	1	no_fusion	2	No	No
CGGA_1495	1	no_fusion	2	#N/A	#N/A
CGGA_1628	1	no_fusion	2	No	#N/A
CGGA_X7	1	no_fusion	#N/A	#N/A	No
CGGA_1766	1	no_fusion	2	#N/A	No
CGGA_773	1	no_fusion	3	No	No
CGGA_X6	1	no_fusion	#N/A	#N/A	No
CGGA_1622	1	no_fusion	3	No	#N/A
CGGA_1625	1	no_fusion	2	Yes	No
CGGA_1585	1	no_fusion	3	No	No
CGGA_1324	1	no_fusion	2	No	No
CGGA_1366	1	no_fusion	2	#N/A	#N/A
CGGA_1197	1	no_fusion	3	Yes	No
CGGA_1734	1	no_fusion	2	Yes	No
CGGA_822	1	no_fusion	2	#N/A	#N/A
CGGA_1186	1	no_fusion	2	No	No
